# Arbovirus-Derived piRNAs Exhibit a Ping-Pong Signature in Mosquito Cells

**DOI:** 10.1371/journal.pone.0030861

**Published:** 2012-01-24

**Authors:** Nicolas Vodovar, Alfred W. Bronkhorst, Koen W. R. van Cleef, Pascal Miesen, Hervé Blanc, Ronald P. van Rij, Maria-Carla Saleh

**Affiliations:** 1 Institut Pasteur, Viruses and RNA interference Group and Centre National de la Recherche Scientifique URA 3015, Paris, France; 2 Department of Medical Microbiology, Radboud University Nijmegen Medical Centre, Nijmegen Centre for Molecular Life Sciences, Nijmegen, The Netherlands; French National Center for Scientific Research - Institut de biologie moléculaire et cellulaire, France

## Abstract

The siRNA pathway is an essential antiviral mechanism in insects. Whether other RNA interference pathways are involved in antiviral defense remains unclear. Here, we report in cells derived from the two main vectors for arboviruses, *Aedes albopictus* and *Aedes aegypti*, the production of viral small RNAs that exhibit the hallmarks of ping-pong derived piwi-associated RNAs (piRNAs) after infection with positive or negative sense RNA viruses. Furthermore, these cells produce endogenous piRNAs that mapped to transposable elements. Our results show that these mosquito cells can initiate *de novo* piRNA production and recapitulate the ping-pong dependent piRNA pathway upon viral infection. The mechanism of viral-piRNA production is discussed.

## Introduction

Arboviruses are maintained in a transmission cycle between hematophagous arthropod vectors and vertebrate hosts. Within their arthropod vector, arboviruses encounter several anatomical and immunological barriers that determine the potential of the virus to be transmitted. RNA interference (RNAi) is a major antiviral defense mechanism in insects [1]-[8]. A hallmark of the insect antiviral RNAi response is the activation of the pathway by cleavage of viral double-stranded RNA (dsRNA) into 21 nucleotides (nt) viral small interfering RNAs (vsiRNA) by Dicer-2 (Dcr-2). Once produced, vsiRNAs guide the sequence-specific recognition and cleavage of viral target RNAs by an Argonaute-2 (AGO-2) containing RNA induced silencing complex.

The siRNA and piRNA (piwi-interacting RNA) pathways are both gene regulatory mechanisms guided by small silencing RNAs in association with an Argonaute family member. piRNAs differ from siRNAs in several aspects [9]: i) piRNAs are generated in a Dicer-independent manner from single-stranded precursors and display a broader size range of ∼25-30 nt; ii) piRNAs associate with the PIWI subclass of the Argonaute family, in flies consisting of piwi, Argonaute-3 (AGO3) and aubergine (aub); iii) PIWI proteins and their associated piRNAs are highly enriched in gonadal tissues, where they protect the germline from activation of transposable elements (TE). Nevertheless, piRNA expression in somatic tissues has recently been reported [10].

Two mechanisms have been proposed for the biogenesis of piRNAs [9]. First, a pool of piRNAs is processed from single-stranded RNA precursors transcribed by chromosomal loci that consist of remnants of TEs. This generates primary piRNAs with a 5′ uridine bias (U_1_) that are usually antisense to TE transcripts. Cleavage of complementary transposon RNA by primary piRNAs initiates the second biogenesis pathway: the ping-pong amplification cycle that involves AGO3 and aub [9]. This amplification loop gives rise to the signature of ping-pong dependent piRNAs: a strong U_1_ bias for aub-associated piRNAs and a bias for adenosine at the tenth position (A_10_) of AGO3-associated piRNAs. PIWI-associated piRNAs have a strong strand bias: AGO3 associates with sense TE piRNAs, whereas piwi and aub associate with antisense TE piRNAs [11], [12].

While the siRNA pathway is well characterized as an antiviral defense mechanism in insects, the involvement of the piRNA pathway has been recently suggested. Indeed, the potential of the primary piRNA pathway to recognize and process viral RNAs was shown in *Drosophila* ovarian somatic sheet cells (OSS cell line) [13] where in addition to the typical Dcr-2 dependent 21 nt siRNAs, a broader peak of ∼25 to 30 nt piRNAs with a U_1_ bias was observed. These cells are capable of producing primary piRNAs but are incapable of ping-pong amplification, due to the lack of aub and AGO3 expression [14]. The existence of viral piRNAs has also been suggested in mosquito, but only based on the size range of the viral small RNAs population [15]-[17].

Here, we show for the first time that mosquito cells infected with (+) and (-) RNA arboviruses produce viral small RNAs with the hallmarks of ping-pong amplification. These results show that mosquito tissue culture faithfully recapitulates the piRNA pathway from an exogenous trigger and may combine RNAi pathways to control a viral infection. These observations have important implications for our understanding of insect innate immunity.

## Results

### Multiple viral small RNAs species in mosquito cells

**Figure 1:**
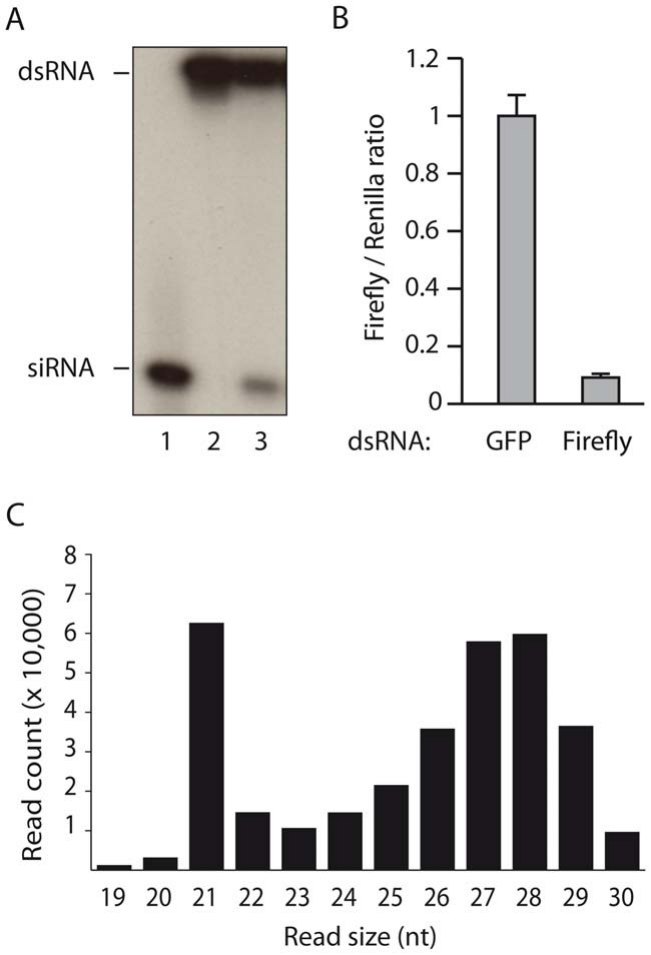
*Aedes albopictus* U4.4 cells are Dcr-2 competent and produce two populations of viral small RNAs. **A.** Dicer assay in uninfected U4.4 cells. Lane 3 shows processing of a 113-bp dsRNA substrate into 21-nt siRNAs after incubation in a U4.4 cell extract. Synthetic siRNA (21-nt) and input dsRNA (113-nt) are used as size markers in lanes 1 and 2, respectively. **B.** RNAi reporter assay. Co-transfection of firefly luciferase specific dsRNA with reporter plasmids encoding firefly and *Renilla* luciferase into U4.4 cells results in silencing of the firefly luciferase reporter. GFP dsRNA was used as non-specific dsRNA control. *Renilla* luciferase activity was used as internal control to normalize the firefly luciferase activity. Error bars represent the standard deviations of three individual samples. **C.** Size distribution of the small RNA reads that match the genome of SINV-GFP with 0 mismatches.

The C6/36 [18] and U4.4 [19] cell lines were cloned from the same cell population isolated from *Aedes albopictus* larvae [20]. C6/36 cells are devoid of Dcr-2 activity [17], but produce virus-derived small RNA that are longer than vsiRNAs, which were proposed to be viral-derived piRNAs (vpiRNAs) [15], [17]. Nevertheless, the absence of functional Dcr-2 activity in C6/36 [17] may have biased these results. To study whether Dcr-2 competent mosquito cells naturally produce vpiRNA, we analyzed viral small RNAs following infection of U4.4 cells. In contrast to C6/36 cells, the U4.4 cells exhibit a functional Dcr-2 activity (Fig. 1). Synthetic ^32^P-labelled dsRNA was effectively processed into 21 nt small RNA in U4.4 cell extracts (Fig. 1A), and dsRNA directed against firefly luciferase efficiently silenced plasmid-driven luciferase expression (Fig. 1B). Altogether, these data show that U4.4 cells possess a functional siRNA pathway that should be able to produce vsiRNAs upon virus infection.

To analyze the impact of Dcr-2 activity on the overall virus-derived small RNA population in *A. albopictus* cells, we infected U4.4 cells with Sindbis virus (SINV), a (+) RNA arbovirus, expressing GFP as a reporter of viral replication. Small RNAs ranging from 19 to 30 nt in length were recovered from infected cells and deep sequenced. Consistent with the Dcr-2 activity detected, the size distribution of virus-derived small RNAs displayed a sharp peak at 21 nt (Fig. 1C) that corresponds to vsiRNAs. In addition, a broader Gaussian distribution that peaks at 27–28 nt was observed (Fig. 1C), which has previously also been reported in C6/36 cells [15], [17].

### *Aedes albopictus* cells produce vpiRNA through a ping-pong mechanism

**Figure 2:**
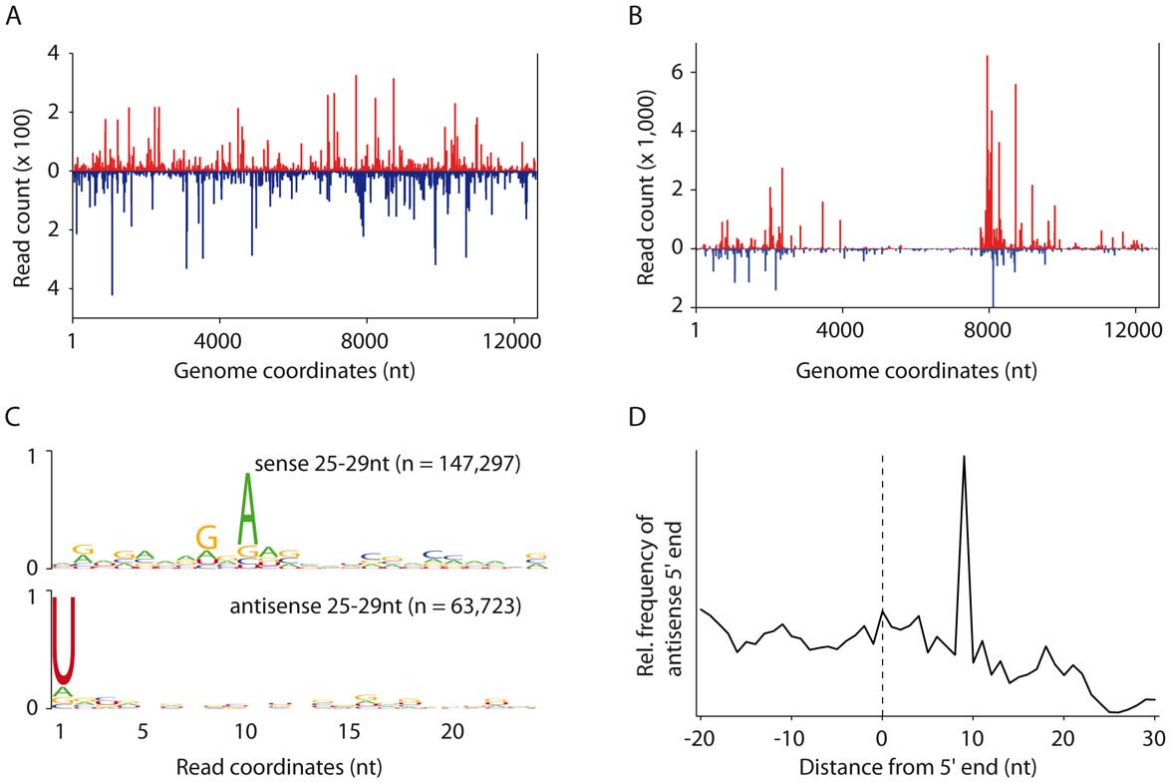
U4.4 cells produce vsiRNAs and vpiRNAs through a ping-pong mechanism upon (+) ssRNA arbovirus infection. Profile of 21 nt vsiRNAs (**A**) and 25–29 nt (**B**) SINV-GFP-derived small RNAs allowing 0 mismatch during alignment. Viral small RNAs that mapped to the sense and antisense strand of the SINV-GFP genome are shown in red and blue, respectively. **C.** Conservation and relative nucleotide frequency per position of 25–29 nt SINV-GFP-derived reads that mapped to the sense (top) and the antisense (bottom) strands of the SINV-GFP genome. The overall height of the nucleotide stack indicates the sequence conservation; the height of the nucleotides within each stack represents their relative frequency at that position. n indicates the number of reads used to generate each logo. **D.** Frequency map of the distance between 25–29 nt small RNAs that mapped to opposite strands of the SINV-GFP genome. The peak at position 9 on the sequence (the first nucleotide being position 0) indicates the position of maximal probability of finding the 5′ end of a complementary small RNA.

We next analyzed the viral small RNA population that peaks at 27–28 nt. Similar to vsiRNAs (Fig. 2A), these small RNAs are distributed across the viral genome, but with an enrichment at the 5′ end of the highly expressed SINV-GFP subgenomic RNA (Fig. 2B). They display a strand bias, with more than 69% of the reads mapping to the sense strand of the viral genome.

OSS cells only produced sense primary vpiRNAs that display a strong U_1_ bias. In contrast, 25 to 29 nt viral small RNAs from SINV-GFP-infected U4.4 cells originate from both viral RNA strands and display the following nucleotide bias (Fig. 2C): vpiRNAs that mapped on the sense strand exhibit a strong A_10_ bias, while vpiRNAs that mapped on the antisense strand displayed a strong U_1_ bias. Furthermore, the 5′ ends of complementary vpiRNAs are most frequently separated by 10 nt (Fig. 2D), which is characteristic of the ping-pong mechanism for piRNA generation [11]. We therefore propose that these viral small RNAs represent ping-pong derived vpiRNAs.

Viral small RNA profiles from SINV-infected C6/36 cells display a similar profile with a size ranging from 19 to 30 nt [15], [17]. We therefore infected C6/36 with SINV-GFP and sequenced the viral small RNA population. Similar to the U4.4 cells, SINV-derived small RNAs from infected C6/36 cells exhibited all the hallmarks of ping-pong amplification (data not shown). Furthermore, the 25–29 nt vpiRNA in C6/36 were resistant to ß-elimination, suggesting that they are associated with a PIWI protein and 2′O methylated at their 3′ terminal nucleotide (Table 1), similar to piRNAs in *Drosophila* and *Bombyx mori* [21], [22]. Altogether, these results show that upon virus infection U4.4 and C6/36 cells produce vpiRNA through a ping-pong amplification mechanism. Furthermore, as C6/36 cells are deficient in Dcr-2 activity, these results suggest that the piRNA pathway is not a backup mechanism when the antiviral siRNA pathway is defective.

**Table 1:** vpiRNAs are resistant to beta-elimination.

	C6/36 + SINV-GFP	C6/36 + SINV-GFP
	No treatment	Beta-elimination
Total number of reads* (19-29nt)	916,504	1,028,574
miRNA reads* (22nt)	286,711	146,225
25–29 nt viral reads	23,737	242,762

*Numbers of reads matching the *Drosophila melanogaster* genome available at flybase and miRNA sequences available at mirBase.

### Ping-pong derived vpiRNAs in (-) RNA virus infection

**Figure 3:**
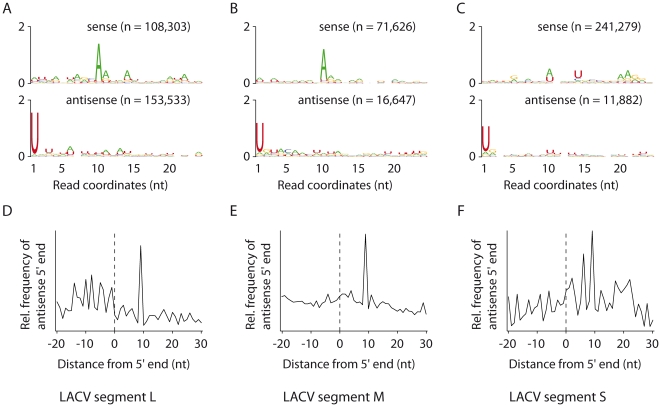
*Aedes albopictus* C6/36 cells produce ping-pong dependent vpiRNA upon (-) RNA virus infection. **A, B**, and **C.** Conservation and relative nucleotide frequency per position of the 25–29 nt LACV-derived reads that mapped to the antigenomic sense (top) and genomic antisense (bottom) strands of the LACV genome segments L, M and S, respectively. n indicates the number of reads used to generate each logo. **D, E**, and **F**. Frequency map of the distance between 25–29 nt reads that mapped to opposite strands of the LACV genome segments L, M and S, respectively. The peak at position 9 on the sequence (the first nucleotide being position 0) indicates the position of maximal probability of finding the 5′ end of a complementary small RNA.

Given the fundamental differences in replication strategies of (+) and (-) RNA viruses, we next analyzed a published dataset from C6/36 cells infected with La Crosse virus (LACV) [15], an arbovirus with a tri-segmented single-stranded (-) RNA genome [23]. The viral RNA segments serve as templates for transcription of viral mRNAs and for the synthesis of full-length viral complementary RNA. Transcripts from the three segments, Large (L), Medium (M) and Small (S), accumulate at different level (S>M>L) [24]. The absolute number of 25–29 nt virus-derived small RNAs did not follow the differential accumulation of each transcript; however, the number of reads normalized for the length of the segments did mirror the much greater mRNA levels of the S segment [20] (S segment 257.3 reads/nt >>L segment 37.5>M segment 19.5). The relative amounts of vpiRNA mapping on each strand of the viral segments differed among the three segments, with ratios of sense over antisense vpiRNAs of 20.3, 4.3, and 0.7 for S, M and L, respectively (Fig. 3A–C). This strand bias of vpiRNA followed the previously estimated gradient of mRNA over viral genome ratios from highly (S) to lower (L) expressed transcripts [20].

Analysis of the nucleotide biases indicated that all segments presented a U_1_ bias on the genomic (−) strand and an A_10_ bias for the antigenomic (+) RNA strand (Fig. 3A–C). In addition, complementary vpiRNAs are enriched for those in which the 5′ ends are separated by exactly 10 nucleotides (Fig. 3D–F). Thus similar to the (+) RNA virus SINV, LACV viral RNAs are targets for ping-pong dependent vpiRNA biogenesis with U_1_ vpiRNAs originating from the negative strand, regardless of viral genome polarity and relative abundance of transcript.

### *Aedes aegypti* Aag2 cells produce vsiRNA and vpiRNA with a ping-pong signature

*A. albopictus* and *A. aegypti* are the major vectors for arboviruses within the *Aedes* genus of culicine mosquitoes. To test whether vpiRNA production also occurs in cells from *A. aegypti*, we analyzed small RNAs in the Aag2 cell line [25] after infection with SINV-GFP.

**Figure 4:**
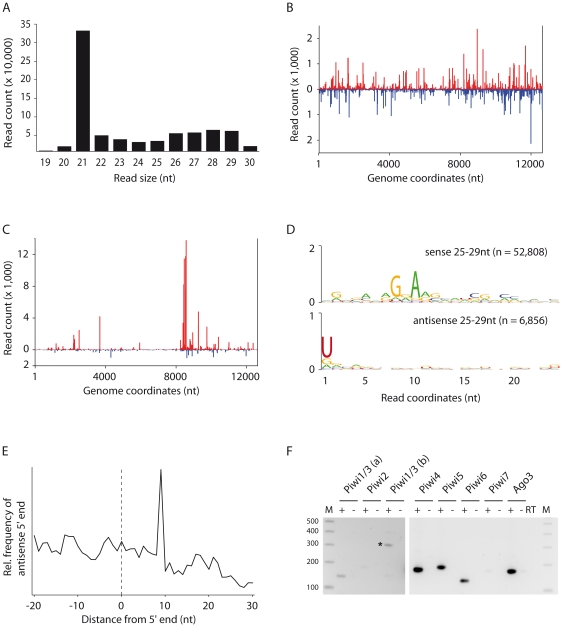
*Aedes aegypti* Aag2 cells produce vsiRNA and vpiRNA with a ping-pong signature upon arbovirus infection. **A.** Size distribution of the small RNA reads that match the genome of SINV-GFP with 0 mismatches. Profile of 21 nt vsiRNAs **(B)** and 25–29 nt **(C)** SINV-GFP-derived small RNAs allowing 0 mismatch during alignment. Viral small RNA that mapped to the sense and antisense strand of the SINV-GFP genome are shown in red and blue, respectively. **D.** Conservation and relative nucleotide frequency per position of 25–29 nt SINV-GFP-derived reads that mapped to the sense (top) and antisense (bottom) strands of the SINV-GFP genome. n indicates the number of reads used to generate each logo. **E.** Frequency map of the distance between 25–29 nt small RNAs that mapped to opposite strands of the SINV-GFP genome. The peak at position 9 on the sequence (the first nucleotide being position 0) indicates the position of maximal probability of finding the 5′ end of a complementary small RNA. **F.** Expression of PIWI family members in Aag2 cells analyzed by RT-PCR. cDNA synthesis was performed in the presence (+) or absence (−) of reverse transcriptase (RT). The -RT samples are included as controls for contamination of RNA preparations with chromosomal DNA. The coding sequences of Piwi1 and Piwi3 are 95% identical at the nucleotide level. Two different primer sets that amplify both Piwi1 and Piwi3 were used (a and b). A higher exposure was used for the gel image with Piwi1to Piwi3. A 100 bp ladder was used as a size marker (M). The asterisk indicates a non-specific PCR amplification product.

We observed a size distribution of virus-derived small RNAs with a sharp peak at 21 nt and a broader Gaussian distribution that peaks at 28 nt (Fig. 4A). Similar to previous observations of Alphavirus infected Aag2 cells [26], the 21 nt vsiRNAs mapped across the viral genome in similar proportions over viral sense and antisense strands (Fig. 4B). The viral small RNAs of 25 to 29 nt are distributed across the viral genome, but enriched at the 5′ end of the highly expressed SINV subgenomic RNA (Fig. 4C). Furthermore, these small RNAs display the hallmarks of ping-pong dependent piRNAs (Fig. 4D–E) as observed in *A. albopictus* cells. Together, our results show that three different cell lines derived from the two major mosquito vectors for arboviruses have a functional PIWI pathway and produce ping-pong derived piRNAs after infection with Sindbis virus.

The PIWI gene family has greatly expanded in *A. aegypti*. In addition to a single *Ago3* orthologue, the *A. aegypti* genome encodes seven *Piwi/Aub* orthologues [27]. Based on their clustering with *Anopheles gambiae Ago4* and *Ago5*, *A. aegypti Piwi1* through *Piwi4* belong to the Ago4 clade, whereas *Piwi5* to *Piwi7* belong to the Ago5 clade. Our observation of ping-pong derived vpiRNAs in mosquito cells implies that PIWI proteins from the different clades are expressed in these cells. Indeed, we readily detected in Aag2 cells transcripts from multiple PIWI family members, including Piwi4, Piwi5, Piwi6, and Ago3 (Fig. 4F).

To address a potential germline source of the Aag2 cells, we analyzed the expression of *Nanos* in Aag2 cells, but we were unable to detect any transcripts by RT-PCR (data not shown). While this result does not rule out a germline origin of the cell line, we do note that the identification of piRNAs with a ping-pong signature in somatic tissues in flies implies that a functional PIWI pathway is present in the soma of insects [10].

### *Aedes aegypti* Aag2 cells produce transposon-derived piRNAs with a ping-pong signature

**Figure 5:**
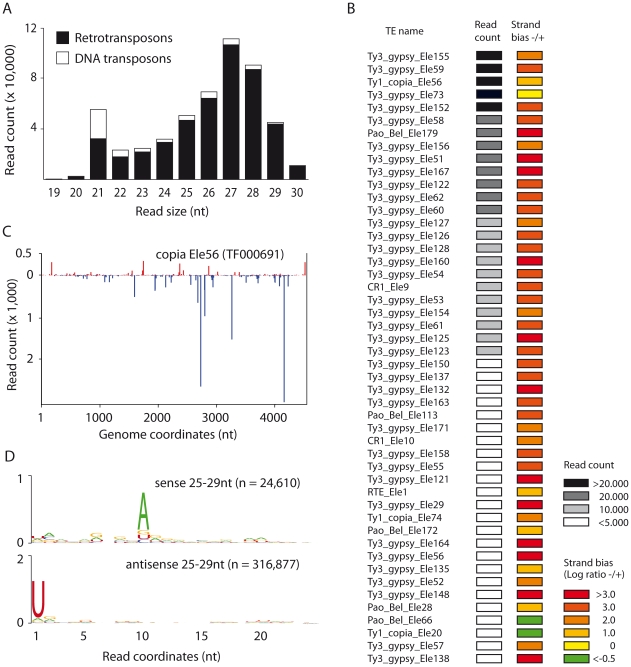
*Aedes aegypti* Aag2 cells produce transposon-derived piRNAs with a ping-pong signature. **A.** Size distribution of the small RNA reads that match with 0 mismatches against an *Aedes aegypti* transposon dataset that contain full-length non-composite transposons sequences (TEfam: http://tefam.biochem.vt.edu/tefam/index.php). **B.** Heat map for 25–29 nt small RNAs that mapped to individual retrotransposons with more than 1000 reads. Read count and log-transformed ratios of antisense/sense small RNAs are presented. **C**. Profile of 25–29 nt reads that mapped to the transposon Copia Ele56 (TF000691) allowing 0 mismatch during alignment. Transposon-derived piRNAs that mapped to the sense and antisense strand of the transposon sequence are shown in red and blue, respectively. **D.** Conservation and relative nucleotide frequency per position of 25–29 nt reads that mapped to the sense (top) and the antisense (bottom) strands of the entire transposon dataset. n indicates the number of reads used to generate each logo.

Our results imply that the piRNA pathway targets replicating RNA viruses in mosquito cells. The majority of piRNAs in *Drosophila* and other animals were described to map to transposable elements. As the genome sequence of *A. aegypti* is available [28], we analyzed whether Aag2 cells engage in ping-pong dependent amplification of TE derived piRNAs. We mapped the non-viral small RNAs to a dataset that contain full-length non-composite transposons sequences (http://tefam.biochem.vt.edu/tefam/index.php). TE-derived small RNAs display a sharp 21 nt peak and a broader peak centering around 27 nt, which is suggestive of TE targeting by the *Aedes* siRNA and piRNA pathways (Fig. 5A). In contrast to TE-derived endo-siRNAs, the vast majority of TE piRNAs derive from retrotransposons and not from DNA transposons (Fig. 5A). For most retrotransposons, the 25–29 nt TE RNAs display a strong over-representation of antisense reads (Fig. 5B–C). The sequence depth of our library did not allow us to analyze ping-pong signatures in individual TEs. We therefore analyzed sequence logos of 25–29 nt small RNAs of the entire retrotransposon dataset (Fig. 5D). A strong U_1_ bias for antisense small RNAs and an enrichment of A_10_ in sense small RNAs imply that, similar to *Drosophila*, TEs are processed by the piRNA pathway in a ping-pong dependent manner in Aag2 cells.

## Discussion

Antiviral RNAi activity in insects has thus far only been attributed to the siRNA pathway. The identification of vpiRNAs in *Drosophila* OSS cells [13] and in *A. aegypti* and *A. albopictus* cells (this study) strongly suggests that the piRNA pathway constitutes another facet of the antiviral RNAi response in insects. Unlike the siRNA pathway, the piRNA pathway is highly enriched in the gonads where it plays a critical role in the control of transposition in the germ line. Because arboviruses can be transmitted vertically in arthropod vectors [29], an antiviral piRNA response in the gonads may constitute an antiviral mechanism to limit vertical transmission of arboviruses in insect vectors. In addition, a putative somatic piRNA pathway may represent an important aspect of vector competence. While the relevance of the piRNA pathway in controlling virus infections awaits experimental validation, it is likely that a pathway that efficiently cleaves viral RNA affects virus replication. Hence, the piRNA pathway should be considered as an intrinsic component of the antiviral RNAi response in insects. Moreover, U4.4 and Aag2 cells emerge as an attractive model to dissect piRNA biogenesis and the interplay between siRNA and piRNA pathways.

Contrary to the OSS cell line that only produces primary vpiRNAs [13], U4.4, Aag2 and C6/36 cells produce primary and secondary vpiRNAs through a ping-pong mechanism. In OSS cells, vpiRNAs map predominantly to the positive strand of the genome of (+) RNA viruses and display the expected U_1_ bias for primary piRNAs. In U4.4, Aag-2 and C6/36 cells however, the nucleotide bias signature is inverted, regardless the polarity of the viral genome. The vpiRNAs that derive from the (−) strand (i.e. the antigenomic strand of SINV and the genomic RNA strand of LACV) present a U_1_ bias, whereas those that derive from the (+) strand display an A_10_ bias. This disparity between OSS cells and mosquito cells is unlikely to be due to differences in piRNA biogenesis, as our results on TE piRNAs in Aag-2 and observations in *Bombyx Mori* BmN4 cells [30] suggest that basic features of piRNA biogenesis are conserved among insects. It is then most likely that this inversion is based on intrinsic features of the viral lifecycle.

The +/− strand ratio is uneven in ssRNA viruses. In (+) RNA viruses, the (+) strand is over-represented compared to the negative strand that serves as template for the production of progeny viral RNA. In many (−) RNA viruses, the (+) viral RNA strand that corresponds to viral transcript is over-represented compared to the genomic (−) strand, although the relative amounts of transcripts are variable. In LACV, there is a gradient of +/− strand ratio between highly (S segment) and slightly (L segment) expressed transcripts. In both (+) and (−) RNA viruses, the genome and the intermediates of replication are shielded from cytoplasmic components, contrary to viral RNAs that engage in translation. Interestingly, primary vpiRNAs are produced from the (−) strand, regardless viral genome polarity. Moreover, in most cases, the ratio between U_1_ and secondary A_10_ vpiRNAs follows strand stoichiometry. According to these observations, we propose two non-mutually exclusive hypotheses for the production of vpiRNAs through a ping-pong mechanism. The first hypothesis is based on the relative amounts of (+) and (−) strands during viral replication. For primary vpiRNAs that are produced from the abundant (+) strand, the generation of secondary vpiRNAs from the (−) strand is limited due to the relative limited amount of viral (−) RNA strands. Conversely, the production of primary vpiRNAs from the (-) strand may allow the generation of abundant secondary vpiRNAs from the abundant (+) strand. According to this hypothesis, as the (+) strand is more abundant than the (−) strand, the second ping-pong mechanism supersedes the first one. As a second hypothesis, the production of primary vpiRNAs from the (−) strand may result for a differential accessibility of the viral RNAs by piRNA pathway components. We propose that the PIWI protein that is responsible for primary piRNA biogenesis can better access viral (−) RNAs, and that the PIWI proteins that are responsible for secondary piRNA biogenesis can mostly access viral (+) RNAs. This may be due to spatial restriction of piRNA pathway proteins or to a differential accessibility of PIWI proteins to the viral RNAs engaged in replication and in translation.

Finally, we show that viruses trigger the piRNA and the siRNA pathways in a similar way as transposons. This suggests that the RNAi pathways only discriminate common features of parasitic nucleic acids rather than their origin.

## Materials and Methods

### Cell culture, virus production and infection

*A. albopictus* U4.4 cells and *A. aegypti* Aag2 cells ([19], [25], kindly provided by G.P. Pijlman, Wageningen University, the Netherlands) were cultured at 28°C in Leibovitz L-15 medium (Invitrogen) supplemented with 10% heat inactivated fetal calf serum (FCS, Invitrogen), 2% Tryptose Phosphate Broth Solution (Sigma) and 1% Non-Essential Amino Acids (Invitrogen). BHK-21 cells (American Type Culture Collection) were cultured in DMEM medium (Invitrogen) supplemented with 10% FCS (Invitrogen), and maintained at 37°C in 5% CO_2_. *In vitro* transcribed RNA from recombinant SINV expressing the Green Fluorescent Protein [31] was transfected into BHK-21 cells. Virus titer was determined by plaque assay on BHK-21 cells. 2×10^6^ U4.4 were infected with SINV-GFP for 2 hours in culture medium at a multiplicity of infection of 1. Cells were harvested 2 days post-infection, when 80-90% of the cells were positive for GFP expression.

### RNAi reporter and Dicer assays

RNAi reporter assays were adapted from [32], using 3×10^5^ U4.4 cells per well of a 24-well plate, 156 ng of pMT-Luc and pMT-Ren plasmids [6], and 0.625 ng of either firefly luciferase or GFP dsRNA. Dicer activity was determined in cell extracts from uninfected U4.4 cells as previously described [33], using 100 counts per seconds of an uniformly ^32^P-radiolabeled 113-bp dsRNA substrate.

### Small RNA library preparation and analysis

Small RNA libraries were prepared as described [34] and sequenced on a Genome Analyzer *IIx* (Illumina). Virus-derived small RNAs were analyzed using Paparazzi [35]. piRNA signatures were calculated using in-house Perl scripts from 25–29 nucleotide-long virus-derived small RNA as previously described [11]. Nucleotide frequencies per position were displayed using the WebLogo program [36]. 19–30 nt reads from the Aag2 small RNA library were aligned with 0 mismatch against the *Aedes aegypti* transposon dataset available at TEfam (http://tefam.biochem.vt.edu/tefam/). The aligned reads were processed similarly to the virus-derived small RNA with in-house Perl scripts. Sequences were submitted to the Sequence Read Archive at the National Center for Biotechnology Information under accession number SRA047263.

### RT-PCR

Total RNA was isolated from a confluent 75 cm^2^ flask of Aag2 cells using Isol-RNA Lysis Reagent (5 Prime) according to manufacturer's recommendations. cDNA synthesis was performed on 1 µg of DNase-I (Invitrogen) treated total RNA using an oligo-dT primer and TaqMan reverse transcriptase (Roche). PCR was performed using the following primers: F-AaeNanos, CAAACGTGAAGCGGAAGATT; R-AaeNanos, AATCAACGATGGATCGGATT; F-AaePIWI1/3a, TGTAGGGGAAGTAATGCATCG; R-AaePIWI1/3a, TCTACGGCAATGGTATCTGCT; F-AaePIWI1/3b, GGCCGTTAGCGAGTCTCAT; R-AaePIWI1/3b, GGCAGAACCTTCGTGGTAAG;; F-AaePIWI2, ATGAAAGCCGGGAAGGTC; R-AaePIWI2, CTGCTACCATTGCCATTTCC; F-AaePIWI4, TGACCGTTACTCTCAAGGGCGCTACCGT; R-AaePIWI4, GACCGTTCACGGCCACCTGCCGAT; F-AaePIWI5, GCCATACATCGGGTCAAAAT; R-AaePIWI5, TGAGGTTGTTGCTTCTGAGGT; F-AaePIWI6, TAATCCACAGGAAGGCTCCA; R-AaePIWI6, CTCCTCCATTGTCCGATCCT; F-AaePIWI7, GGAGGTCGTGGAGGTAACAA; R-AaePIWI7, CCTTCCAATCACGATTGCTT; F-AaeAgo3, TCGGTTTACCGCCAGCTGGGAGTTTTG; R-AaeAgo3, AGGTTATCTCAGCGGGAAAATCATGTCGCT.
